# Supplementary figures and images for: AATF inhibition exerts antiangiogenic effects against human hepatocellular carcinoma
Source: Front Oncol. 2023 Jun 9;13:1130380. doi: 10.3389/fonc.2023.1130380 (PMC10288852; doi:10.3389/fonc.2023.1130380)

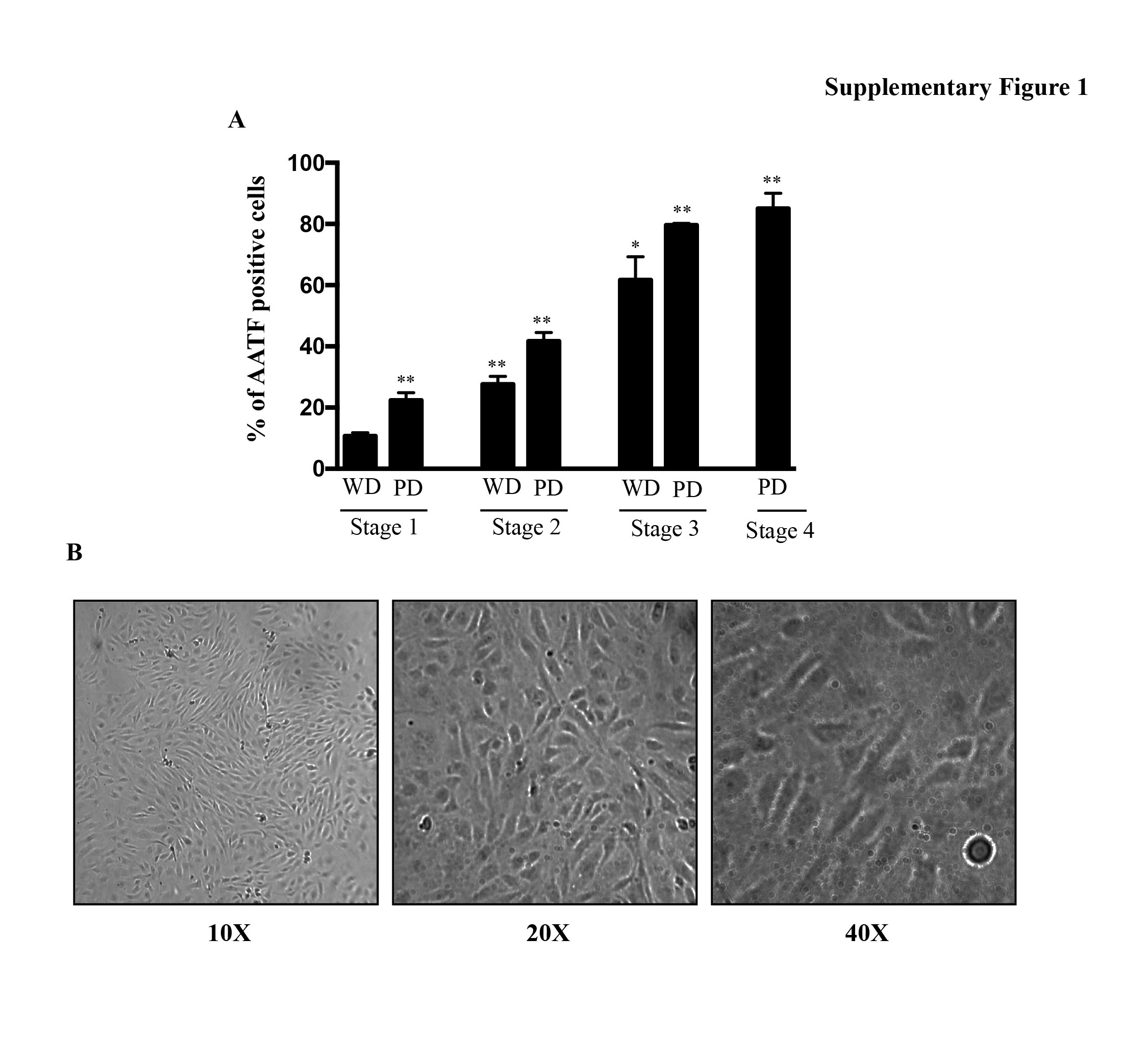

Supplement: Supplementary Figure 1 — (A) Quantitation and comparison of the AATF-positive cells in HCC tissues of stage 1, stage 2, stage 3 and stage 4 using Image J software. WD, well differentiated; PD, poorly differentiated HCC tissues. (B) Culturing of Human umbilical vein endothelial cells (HUVECs). Images at different magnifications- 10X, 20X and 40X. [file Image_1.jpg]

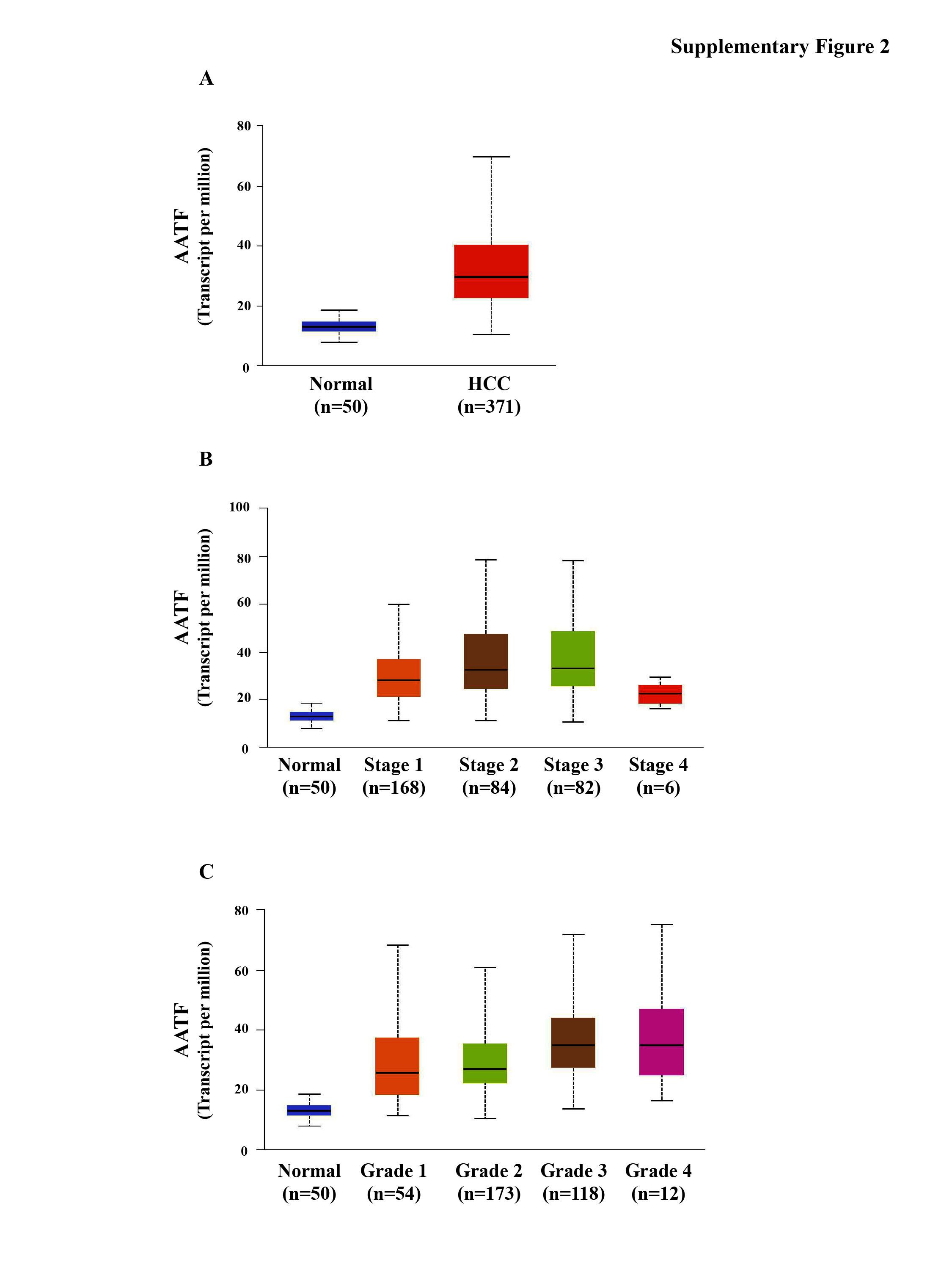

Supplement: Supplementary Figure 2 — (A) Differential expression Analysis of AATF in normal (n=50) and human HCC (n=371) tissues in TCGA microarray data set (P<1x10^-12^). (B) Expression of AATF varies across the stages of HCC (normal vs. stage 1: P<1.62x10^-12^; normal vs. stage 2: P<1.62x10^-12^; normal vs. stage 3: P<1x10^-12^; normal vs. stage 4: P<7.7x10^-2^). (C) Tumor grades were highly significant (normal vs. grade 1: P<1.33x10^-10^; normal vs. grade 2: P<1x10^-12^; normal vs. grade 3: P<1x10^-12^; normal vs. grade4: P<6.4x10^-4^). [file Image_2.jpg]

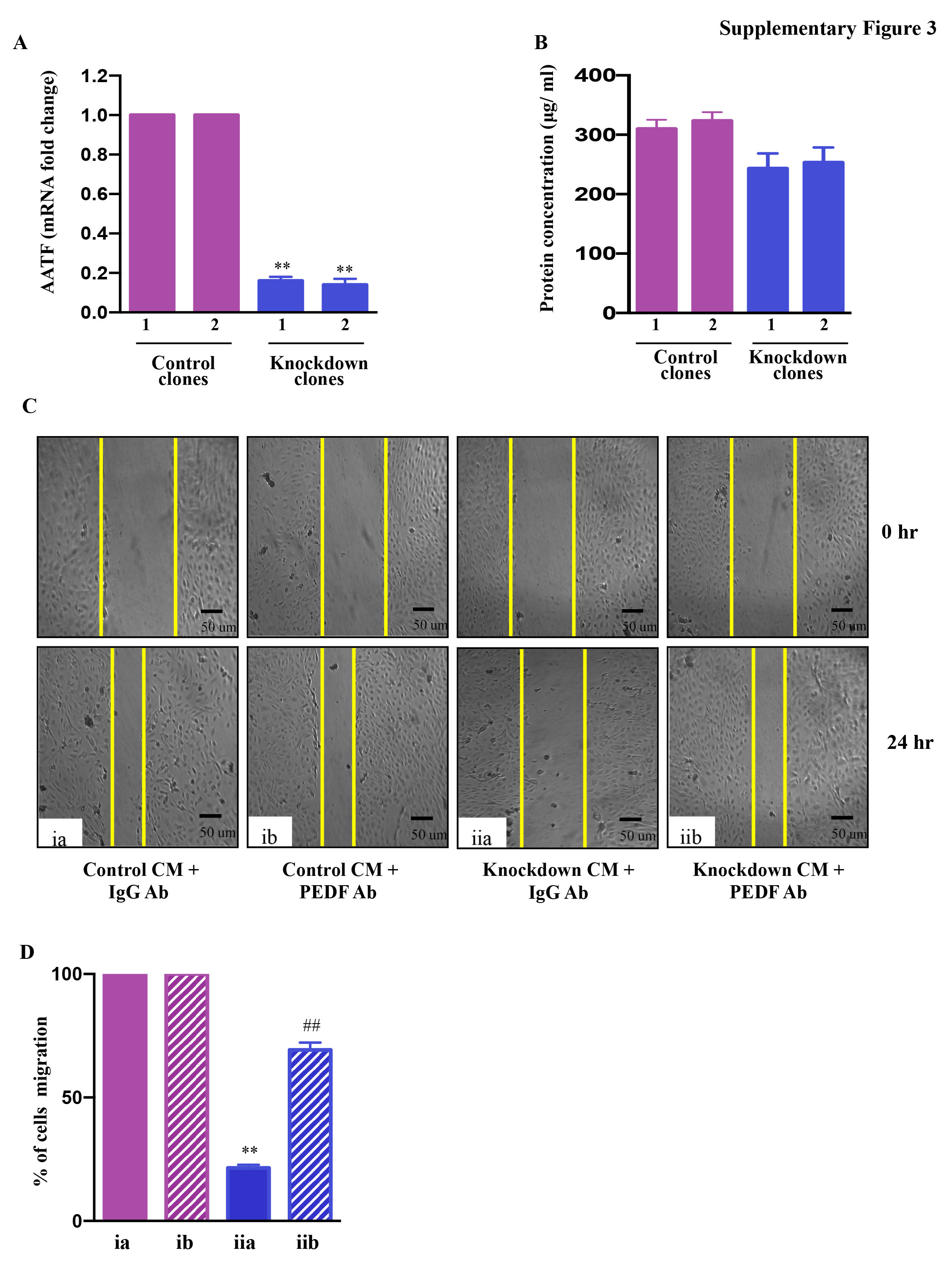

Supplement: Supplementary Figure 3 — (A) AATF mRNA expression in control (clone 1 and clone 2) and AATF knockdown (clone 1 and clone 2) QGY- 7703 cells. (B) Protein concentration (μg/ml) of conditioned media from control and (clone 1 and clone 2) and AATF knockdown (clone 1 and clone 2) QGY- 7703 cells. (C) Effect of conditioned media from control (clone 2) and AATF knockdown (clone 2) QGY-7703 cells treated with or without anti-PEDF antibody on migration (D) Quantification of the gap distance at 0 hr and 24 hr was evaluated using Image J software and expressed as % cells migration. Data are expressed as the mean ± SEM of three experiments. **p < 0.001 or *p < 0.05 compared to control. [file Image_3.jpg]
