# Supplementary material for: AATF inhibition exerts antiangiogenic effects against human hepatocellular carcinoma
Source: Front Oncol. 2023 Jun 9;13:1130380. doi: 10.3389/fonc.2023.1130380 (PMC10288852; doi:10.3389/fonc.2023.1130380)
Supplement: Supplementary Table 1 — Demographic and clinicopathological data of HCC patients. M, male; F, female; HCC, hepatocellular carcinoma; TNM, tumor, nodes and metastases; PD, poorly differentiated; MD, moderately differentiated; WD, well differentiated. [file DataSheet_1.pdf]

Supplementary Table 1

Sl. No.	Gender	Age (yr)	Patient Diagnosis	TNM	Tumor Stage	Tumor Grade
1.	F	38	HCC	pT1bNxM0	Stage 1	PD
2.	M	78	HCC	pT1bN0M0	Stage 1	MD
3.	M	64	HCC	pT1bNxM0	Stage 1	MD
4.	F	35	HCC, infiltrating stomach	pT4NxM0	Stage 4	MD
5.	F	56	HCC of left lobe of liver	pT3N1M0	Stage 3	MD
6.	M	81	HCC	pT3N0M0	Stage 3	MD
7.	M	43	HCC	pT1bNxM0	Stage 1	MD
8.	F	72	HCC	pT2N0M0	Stage 2	MD
9.	M	55	HCC	pT1bNxM0	Stage 1	MD
10.	F	49	HCC	pT1bNxM0	Stage 1	MD
11.	M	85	HCC	pT1bNxM0	Stage 1	MD
12.	F	30	HCC	pT3aN0MX	Stage 3	MD
13.	F	70	HCC	pT3NxM0	Stage 3	PD
14.	F	67	HCC	pT3N0M0	Stage 3	WD
15.	M	72	HCC	pT3NxM0	Stage 3	PD
16.	M	42	HCC	pT3N0M0	Stage 3	PD
17.	M	46	HCC	pT2NxM0	Stage 2	PD
18.	M	81	HCC	pT3NxM0	Stage 3	MD
19.	M	65	HCC	pT1bN0M0	Stage 1	MD
20.	M	69	HCC	pT3N0M0	Stage 3	MD
21.	M	44	HCC	pT2NxM0	Stage 2	PD

22.	M	67	HCC	pT2NxM0	Stage 2	MD
23.	M	51	HCC	pT4N0	Stage 4	MD
24.	M	35	HCC	pT2NxM0	Stage 2	MD
25.	M	66	HCC	pT1NxMx	Stage 1	MD
26.	M	59	HCC	pT4NxM0	Stage 4	PD
27.	M	68	HCC, anaplastic type	pT3NxM0	Stage 3	PD
28.	M	51	HCC	pT2NxM0	Stage 2	MD
29.	M	67	HCC	pT2N0M0	Stage 2	MD
30.	M	46	HCC	pT1bN0M0	Stage 1	MD
31.	M	73	HCC	pT2NxM0	Stage 2	MD
32.	F	52	HCC	pT3NxM0	Stage 3	PD
33.	M	80	HCC	pT1bNxM0	Stage 1	MD
34.	M	69	HCC	pT3N0M0	Stage 3	MD
35.	M	54	HCC	pT1bNxM0	Stage 1	PD
36.	F	44	HCC	pT2NxM0	Stage 2	MD
37.	M	53	HCC	pT2NxM0	Stage 2	PD
38.	F	61	HCC	pT3N0M0	Stage 3	MD
39.	M	77	HCC	pT2	Stage 2	PD
40.	M	71	HCC with predominant undifferentiated morphology	pT3NxM0	Stage 3	PD
41.	M	71	HCC	T3N0M0	Stage 3	PD
42.	F	54	HCC	pT1bN0M0	Stage 1	MD
43.	M	49	HCC	pT3NxM0	Stage 3	MD

44.	M	75	HCC	pT2NxM0	Stage 2	PD
45.	M	25	HCC, fibrolamellar	pT3NxM0	Stage 3	PD
46.	M	51	HCC	pT2N0	Stage 2	WD
47.	M	54	HCC	pT1NxM0	Stage 1	WD
48.	M	72	HCC	pT3NXMX	Stage 3	MD
49.	F	36	HCC	pT1bN0M0	Stage 1	MD
50.	M	45	HCC	pT2pN0pM0	Stage 2	MD
