# Supplementary material for: AATF inhibition exerts antiangiogenic effects against human hepatocellular carcinoma
Source: Front Oncol. 2023 Jun 9;13:1130380. doi: 10.3389/fonc.2023.1130380 (PMC10288852; doi:10.3389/fonc.2023.1130380)
Supplement: Supplementary Table 2 — List of primers used in qRT-PCR. [file DataSheet_2.pdf]

Supplementary Table 2

Name	Forward Primer	Backward Primer
**AATF**	TAGAACGGAAGACCAGCTCC	TGCTAAGGACATGAAACCGA
**β- actin**	AGAGATGGCCACGGCTGCTT	CAGGACTCCATGCCCAGGAA
**PEDF**	GGTGCAGGCGCAGATGAAAG	TTGTTACCCACTGCCCCTTGA
**MMP2**	TACGATGGAGGCGCTAATGGC	GAAGGTGTTCAGGTATTGCACTG
**MMP9**	TCTGCCCGGACCAAGGATAC	CCCCTCAGTGAAGCGGTACAT
